# Racial, Ethnic, and Sex Diversity Trends in Health Professions Programs From Applicants to Graduates

**DOI:** 10.1001/jamanetworkopen.2023.47817

**Published:** 2023-12-28

**Authors:** Daniel Majerczyk, Erin M. Behnen, David J. Weldon, Roy Kanbar, Yolanda M. Hardy, Stanley K. Matsuda, Karen L. Hardinger, Farid G. Khalafalla

**Affiliations:** 1 Department of Family Medicine, Loyola Medicine–MacNeal Family Medicine Residency Program, Berwyn, Illinois; 2 College of Science, Health and Pharmacy, Roosevelt University, Schaumburg, Illinois; 3 Harvard Graduate School of Education, Harvard University, Cambridge, Massachusetts; 4 College of Pharmacy, Belmont University, Nashville, Tennessee; 5 School of Pharmacy, William Carey University, Biloxi, Mississippi; 6 School of Pharmacy, Lebanese American University, Byblos, Lebanon; 7 Lloyd L. Gregory School of Pharmacy, Palm Beach Atlantic University, West Palm Beach, Florida; 8 School of Pharmacy, Loma Linda University, Loma Linda, California; 9 School of Pharmacy, University of Missouri-Kansas City, Kansas City; 10 College of Education and Health Sciences, Touro University California, Vallejo

## Abstract

**Importance:**

Diversity is an essential element of an effective health care system. A key to developing a diverse workforce is establishing a diverse student population in health professions programs.

**Objective:**

To examine the diversity of students in Doctor of Medicine (MD), Doctor of Osteopathic Medicine (DO), Doctor of Dental Surgery (DDS), Doctor of Dental Medicine (DMD), and Doctor of Pharmacy (PharmD) programs with emphasis on the trends of underrepresented minoritized groups (American Indian or Alaska Native, Black or African American, Hispanic or Latino, and Native Hawaiian or Other Pacific Islander) and sex relative to the overall age-adjusted US population.

**Design, Setting, and Participants:**

This cross-sectional study used deidentified, self-reported data from 2003 to 2019 from the Association of American Medical Colleges, American Association of Colleges of Osteopathic Medicine, American Dental Education Association, American Dental Association, and American Association of Colleges of Pharmacy. Data analysis was performed from 2003 to 2004 and from 2018 to 2019.

**Exposures:**

Data on the race, ethnicity, and sex of applicants, matriculants, and degrees conferred by health professions programs were collected and compared with the age-adjusted population in the US Census (aged 20-34 years) over time.

**Main Outcomes and Measures:**

The main outcomes were trends in the proportions of underrepresented minoritized groups and sex diversity among applicants, matriculants, and degrees conferred relative to the overall age-adjusted US population. Trends were measured using the representation quotient, which is defined as the ratio of the proportion of each subgroup to the total population of applicants, matriculants, or graduates relative to the proportion for that subgroup within the US Census population of similar age. Regression analysis was used to evaluate the trend over time.

**Results:**

A total of 594 352 applicants were analyzed across the examined programs. From 2003 to 2019, the proportions of individuals from underrepresented groups increased for DDS and DMD (applicants, from 1003 of 8176 to 1962 of 11 298 [5.1%]; matriculants, from 510 of 4528 to 966 of 6163 [4.2%]; degrees awarded, from 484 of 4350 to 878 of 6340 [2.7%]), PharmD (applicants, from 9045 of 71 966 to 11 653 of 50 482 [9.0%]; matriculants, from 5979 of 42 627 to 10 129 to 62 504 [6.3%]; degrees awarded, from 922 of 7770 to 2190 of 14 800 [3.0%]), and DO (applicants, from 740 of 6814 to 3478 of 21 090 [5.4%]; degrees awarded, 199 of 2713 to 582 of 6703 [1.4%]) programs, but decreased for MD programs (applicants, from 6066 of 34 791 to 7889 of 52 777 [−2.3%]; matriculants, 2506 of 16 541 to 2952 of 21 622 [−2.4%]; degrees awarded, from 2167 of 15 829 to 2349 of 19 937 [−0.1%]). Compared with age-adjusted US Census data, all programs had more Asian students and fewer male, American Indian or Alaska Native, Black or African American, Hispanic or Latino, and Native Hawaiian or Other Pacific Islander students (representation quotient <1).

**Conclusions and Relevance:**

In this cross-sectional study, most of the health professions in the study saw increases in underrepresented minority applicants, matriculants, and degrees conferred from 2003 to 2019; however, all programs were below the age-adjusted US Census data. The increased racial, ethnic, and sex diversity in the programs illustrates progress, but additional strategies are needed to achieve a more representative health care workforce.

## Key Points

**Question:**

Does the racial and ethnic and sex diversity of students in 4 US health professions programs parallel the diversity of the age-adjusted US population?

**Findings:**

This cross-sectional analysis of applicant, matriculant, and degrees conferred data in medicine, dentistry, and pharmacy programs for 594 352 students between 2003 and 2019 found an increase in underrepresented minoritized groups in most health professions programs and a lower percentage of male students compared with age-adjusted US Census data.

**Meaning:**

These findings suggest that progress has been made to increase racial, ethnic, and sex diversity among students in most health professions programs, but additional strategies are needed to achieve a more representative health care workforce.

## Teaser

This cross-sectional study examines the diversity of students in health profession programs with an emphasis on the trends of underrepresented minority groups and sex relative to the overall age-adjusted US population.

## Introduction

Increasing diversity remains a common goal for all health care–related degree programs because it may reduce health disparities, improve health care delivery, and meet the needs of an increasingly diverse population.^1,2^ Over the past 2 decades, health care practitioners, public health professionals, and policy makers have proposed collaborative models to promote diversity in the health care workforce. In 2004, experts in health care, business, higher education, and law released the *Sullivan Report on Diversity in the Healthcare Workforce*,^3^ which highlighted lack of diversity as a major cause of health care disparities in access and patient outcomes. The report advocated for a workforce more closely mirroring the population it serves.^3^ Similarly, in 2015, congressional representatives, medical professionals, and minority health leaders published a report^4^ on health disparities that urged retooling health parity by focusing on key areas of workforce diversity.

A diverse health care workforce enhances patient care by addressing unique cultural, social, and linguistic needs.^5^ Racial concordance between patients and health care practitioners increases the likelihood of patient visits,^6^ enhances communication effectiveness,^7^ improves patient outcomes,^8,9^ and reduces health disparities in community pharmacy settings.^10^

As the US becomes increasingly diverse, the need for diversity in health care intensifies.^11^ Over the past 15 years, racial and ethnic diversity in various US regions increased by 1% to 5% on average.^12^ A key to a diverse workforce is a diverse student population in health professions programs, which is strongly influenced by cultural, socioeconomic, and legal factors, including the recent US Supreme Court ruling on using race in college admissions.^13,14,15,16,17,18^ Diversity within the student population yields benefits at individual, organizational, and societal levels. Formal classroom and informal campus interactions in a diverse atmosphere are associated with improved learning outcomes, particularly in active thinking skills, intellectual engagement, and motivation.^19^ Diverse learning environments also positively impact student behavior and mindset and further psychosocial development by reinforcing understanding, respect, and appreciation of various cultures, personal lifestyles, and professional experiences.^19,20,21,22^ Cultural competency and intellectual skills acquired in diverse learning environments nurture effective communication and teamwork, which are crucial assets for patient-centered collaborative practice.^19,22,23^

The objective of this study is to examine the underrepresented minority (URM) and sex diversity of applicants, matriculants, and degrees conferred to students within the Doctor of Medicine (MD), Doctor of Osteopathic Medicine (DO), Doctor of Dental Surgery (DDS), Doctor of Dental Medicine (DMD), and Doctor of Pharmacy (PharmD) degree programs in the US relative to the overall age-adjusted US population. With this report, we provide a systematic approach to assessing the representation of medical, dental, and pharmacy school applicants, matriculants, and graduates relative to the racial, ethnic, and sex distribution of the age-adjusted US population.

## Methods

Deidentified, self-reported race, ethnicity, and sex data from the Association of American Medical Colleges,^24^ American Association of Colleges of Osteopathic Medicine (complete data are available only after 2008),^25^ American Dental Education Association,^26^ American Dental Association,^27^ and American Association of Colleges of Pharmacy (matriculant data unavailable from 2003-2004)^28^ were analyzed and compared with national age-adjusted US Census data (ages 20-34 years).^12^ Data regarding applications, matriculated students, and degrees conferred across health care professions were collected from 2003 to 2019. Study variables included applicants, matriculants, and graduates by URM individuals, stratified by race, ethnicity, and sex. Study variables were compared with age-adjusted US Census data. A member of a URM group was defined as self-identified American Indian or Alaska Native, Black or African American, or Hispanic or Latino students who are US citizens or permanent residents. Beginning in 2011, Native Hawaiian or Other Pacific Islander students were also included. The analysis was considered exempt from review and the need for informed consent by the William Carey University institutional review board because the data were publicly available and anonymous, in accordance with 45 CFR §46. We followed the Strengthening the Reporting of Observational Studies in Epidemiology (STROBE) reporting guideline.

### Statistical Analysis

Data analysis was performed from 2003 to 2004 and from 2018 to 2019. Descriptive statistics were used to examine trends in the percentage of individuals from URM groups, race, ethnicity, and sex among students applying to, matriculating into, and graduating from health professions programs, compared with a similar age group in the US Census population. A representation quotient (RQ) was applied.^29^ The RQ is the ratio of the proportion of each subgroup to the total population of applicants, matriculants, or graduates relative to the proportion for that subgroup within the US Census population of similar age. An RQ of 1 indicates that the proportion of the subgroup is the same as the proportion for that subgroup within the US Census population of similar age. An RQ less than 1 demonstrates lower subgroup representation than the proportion for that subgroup within the US Census population of similar age. An RQ exceeding 1 demonstrates higher representation than the US Census population of similar age.^29^ Each subgroup’s longitudinal trends on RQ (regression analysis performed with Excel version 2019, Microsoft) are reported over time with corresponding 2-sided *P* values and 95% CIs, with statistical significance set at *P* < .05.

## Results

### Applicant Overview

This study analyzed 594 352 applicants across MD, DO, DDS/DMD, and PharmD degree programs for academic years of 2003 to 2004, 2008 to 2009, 2013 to 2014, and 2018 to 2019. Table 1 summarizes the total number of applicants, matriculants, and degrees conferred. MD programs had the greatest number in all categories, followed by PharmD; DO and DDS/DMD had similar numbers of matriculants and degrees conferred.

**Table 1.** Participants in Health Degree Programs in 2018 and 2019

Category	Participants, No.			
	Doctor of Medicine	Doctor of Osteopathic Medicine	Doctor of Dental Surgery/Doctor of Dental Medicine	Doctor of Pharmacy
Applicants	53 370	21 090	11 298	50 842
Matriculants	21 869	7372	6250	12 795
Degrees conferred	19 935	6703	6305	14 800

### URM Representation

**Figure 1.**
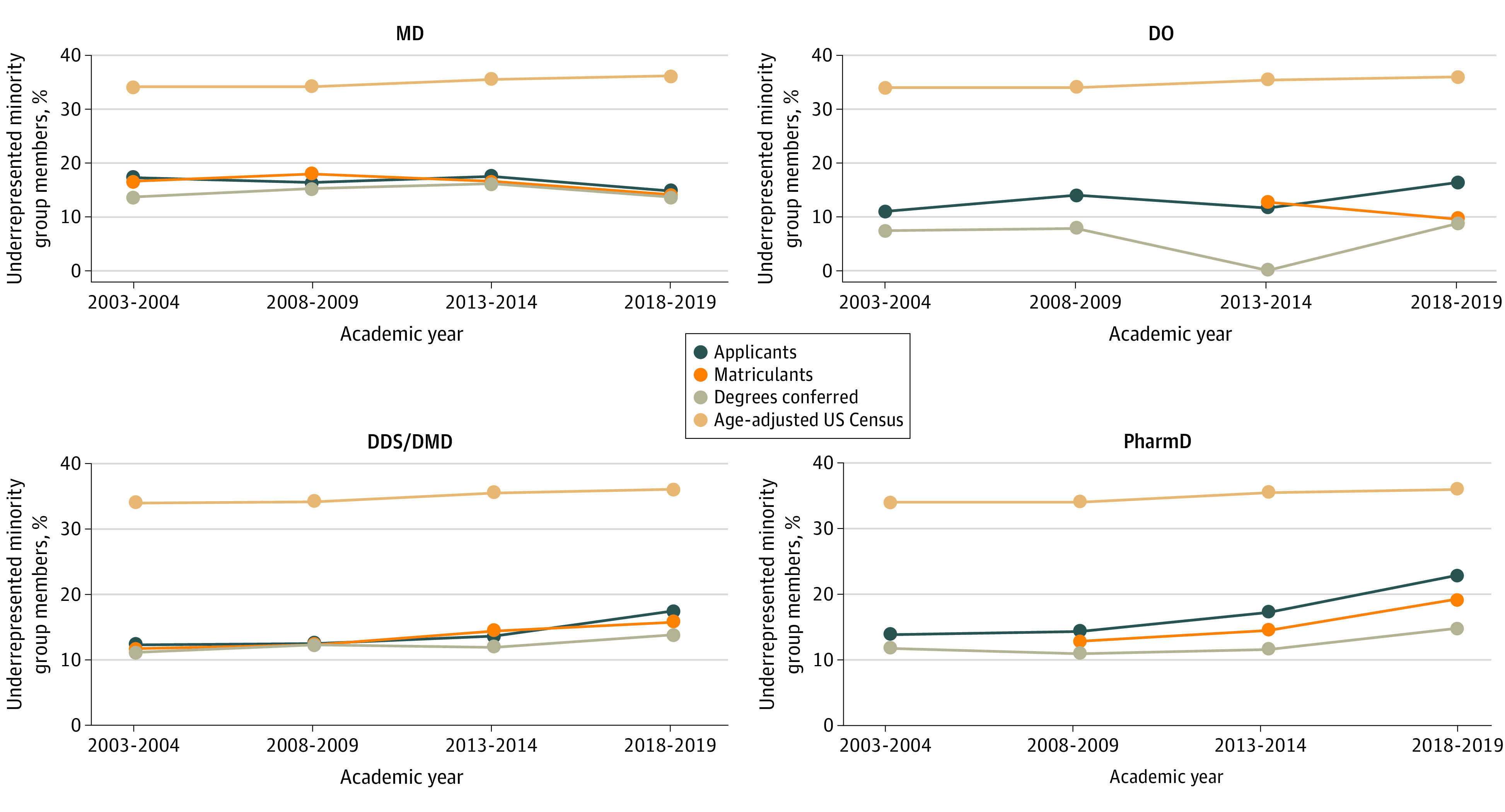
Individuals From Underrepresented Minority Groups in 4 Health Degree Programs, 2003-2019 Graphs show percentages of underrepresented minority group (American Indian or Alaska Native, Black or African American, Hispanic or Latino, and Native Hawaiian or Other Pacific Islander) members in Doctor of Medicine (MD), Doctor of Osteopathic Medicine (DO), Doctor of Dental Surgery/Medicine (DDS/DMD) and Doctor of Pharmacy (PharmD) programs compared with the overall US population.

Although all 4 health degree programs were below the percentage of URM population nationally according to age-adjusted data from the US Census (Figure 1), the percentage of URM applicants and graduates increased from 2003 to 2019 in 3 of the 4 programs (DDS and DMD, applicants, from 1003 of 8176 to 1962 of 11 298 [5.1%]; matriculants, from 510 of 4528 to 966 of 6163 [4.2%]; degrees awarded, from 484 of 4350 to 878 of 6340 [2.7%]; PharmD, from 9045 of 71 966 to 11 653 of 50 482 [9.0%]; matriculants, from 5979 of 42 627 to 10 129 to 62 504 [6.3%]; degrees awarded, from 922 of 7770 to 2190 of 14 800 [3.0%]; and DO, applicants, from 740 of 6814 to 3478 of 21 090 [5.4%]; degrees awarded, 199 of 2713 to 582 of 6703 [1.4%]). DO programs had decreases in URM matriculants between 2013 and 2019 by 3.0% (previous years’ data were lost during a physical move and electronic migration of records, according to American Association of Colleges of Osteopathic Medicine). MD programs experienced a decrease in URM students in all 3 areas (applicants, from 6066 of 34 791 to 7889 of 52 777 [−2.3%]; matriculants, 2506 of 16 541 to 2952 of 21 622 [−2.4%]; degrees awarded, from 2167 of 15 829 to 2349 of 19 937 [−0.1%]). Figure 1 shows the trends for each program.

### Race and Ethnicity Distribution

**Figure 2.**
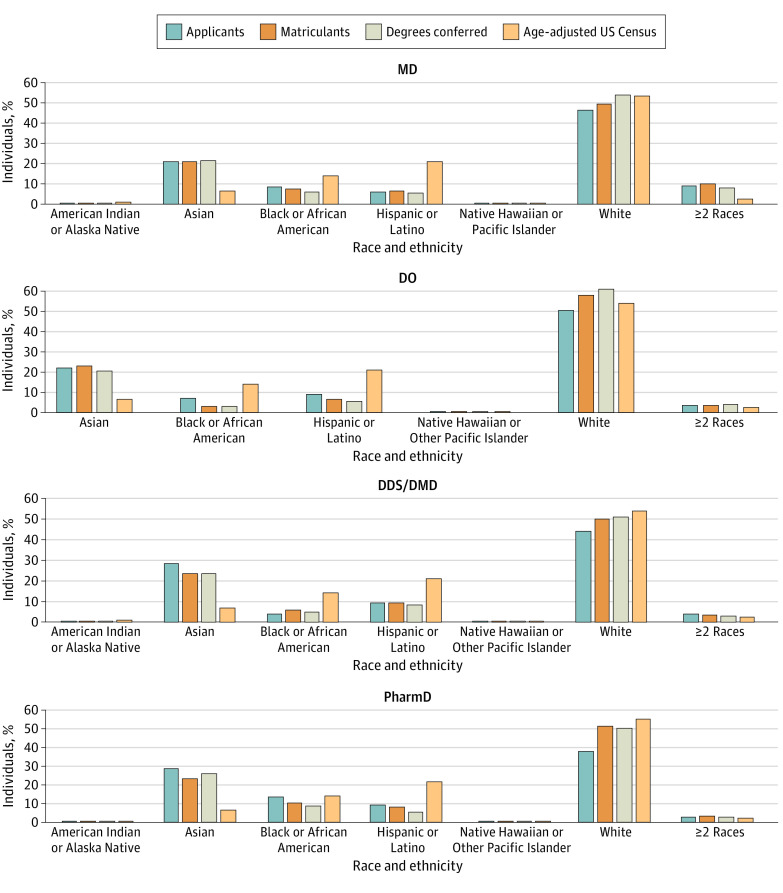
Percentages of Individuals From Underrepresented Minority Groups in 4 Health Degree Programs by Racial and Ethnic Category, 2018 and 2019 Graphs show percentages of underrepresented minority group members in Doctor of Medicine (MD), Doctor of Osteopathic Medicine (DO), Doctor of Dental Surgery/Medicine (DDS/DMD), and Doctor of Pharmacy (PharmD) programs compared with the overall US population.

Each race and ethnicity category was analyzed within each program in 2018 to 2019 and compared with the US Census data (Figure 2). All 4 health professions programs had considerably more Asian applicants, matriculants, and graduates compared with age-adjusted US Census data. DDS/DMD and PharmD programs had fewer White students in all 3 categories compared with the age-adjusted 2020 US Census, whereas MD and DO programs had more degrees conferred for White students.

### Representation Quotient

The percentage of American Indian or Alaska Native, Black or African American, Hispanic or Latino, and Native Hawaiian or Other Pacific Islander applicants, matriculants, and graduates in each program were below their respective national percentages. Similar to the trend data, the RQ for total URM students for all 4 programs was 1 or less for applicants, matriculants, and degrees conferred in 2018 and 2019 compared with age-adjusted US Census data (Table 2; eFigure 1 in Supplement 1). In 2019, MD degrees conferred to students from URM groups had an overall RQ of 0.33. Of all URM groups, Black or African American students had the highest RQ in MD programs (0.43). DO degree conferral representation had an even lower RQ (0.24) for students from URM groups in 2019. The RQ for Black or African American representation was lowest (0.21). Similarly, the RQs for degree conferral in DDS/DMD and PharmD programs were 0.38 and 0.41, respectively (eFigure 2 in Supplement 1).

**Table 2.** Slope of RQ Over Time, 2003-2019

Program and race, ethnicity, and gender category	RQ estimate (95% CI)^a^	*P* value
Applicants		
MD		
American Indian or Alaskan Native	−0.0732 (−0.1651 to 0.0188)	.08
Black or African American	−0.0056 (−0.0187 to 0.0074)	.20
Hispanic or Latino	−0.0064 (−0.0228 to 0.0100)	.24
Native Hawaiian or other Pacific Islander	−0.0974 (−0.3368 to 0.1420)	.22
Total underrepresented minorities^b^	−0.0087 (−0.0188 to 0.0014)	.06
Male	−0.0008 (−0.0174 to 0.0159)	.86
DO		
American Indian or Alaskan Native	−0.0427 (−0.1521 to 0.0668)	.24
Black or African American	0.0048 (−0.0224 to 0.0320)	.53
Hispanic or Latino	0.0074 (−0.0261 to 0.010)	.44
Native Hawaiian or other Pacific Islander	0.0131 (−0.848 to 0.1110)	.62
Total underrepresented minorities^b^	0.0049 (−0.0171 to 0.0270)	.44
Male	−0.0025 (−0.0140 to 0.0090)	.44
DDS/DMD		
American Indian or Alaskan Native	−0.0459 (−0.0887 to −0.0032)	.04
Black or African American	−0.0027 (−0.0203 to −0.0149)	.58
Hispanic or Latino	0.0140 (−0.0155 to 0.0434)	.18
Native Hawaiian or other Pacific Islander	Not enough data	NA
Total underrepresented minorities^b^	0.0059 (−0.0158 to 0.0276)	.37
Male	−0.0123 (−0.0147 to −0.0098)	.002
PharmD		
American Indian or Alaskan Native	−0.0274 (−0.0497 to −0.0050)	.03
Black or African American	0.0187 (0.0007 to 0.0366)	.046
Hispanic or Latino	0.0155 (−0.0075 to 0.0385)	.10
Native Hawaiian or other Pacific Islander	Not enough data	NA
Total underrepresented minorities^b^	0.0154 (−0.0034 to 0.0341)	.07
Male	−0.0005 (−0.0151 to 0.0142)	.90
Matriculants		
MD		
American Indian or Alaskan Native	−0.0671 (−0.1727 to 0.0384)	.11
Black or African American	−0.0044 (−0.0105 to 0.0016)	.09
Hispanic or Latino	−0.0042 (−0.0296 to 0.0212)	.55
Native Hawaiian or other Pacific Islander	−0.0546 (−0.2211 to 0.1119)	.29
Total underrepresented minorities^b^	−0.0064 (−0.0217 to 0.0088)	.21
Male	−0.0034 (−0.0173 to 0.0105)	.40
DO		
American Indian or Alaskan Native	Not enough data	NA
Black or African American	Not enough data	NA
Hispanic or Latino	Not enough data	NA
Native Hawaiian or other Pacific Islander	Not enough data	NA
Total underrepresented minorities^b^	Not enough data	NA
Male	Not enough data	NA
DDS/DMD		
American Indian or Alaskan Native	−0.0393 (−0.1839 to 0.1052)	.36
Black or African American	−0.0060 (−0.0215 to 0.0095)	.24
Hispanic or Latino	0.0136 (−0.0004 to 0.0276)	.05
Native Hawaiian or other Pacific Islander	Not enough data	NA
Total underrepresented minorities^b^	0.0045 (−0.0063 to 0.0152)	.22
Male	−0.0109 (−0.0196 to −0.0022)	.03
PharmD		
American Indian or Alaskan Native	−0.0114 (−0.0416 to 0.0188)	.25
Black or African American	−0.0062 (−0.0625 to 0.0501)	.68
Hispanic or Latino	0.0055 (−0.0110 to 0.0220)	.29
Native Hawaiian or other Pacific Islander	Not enough data	NA
Total underrepresented minorities^b^	0.0001 (−0.0327 to 0.0329)	.99
Male	0.0040 (−0.0115 to 0.0195)	.38
Degrees conferred		
MD		
American Indian or Alaskan Native	−0.0332 (−0.1716 to 0.1051)	.41
Black or African American	−0.0044 (−0.0077 to −0.0011)	.03
Hispanic or Latino	−0.0064 (−0.0316 to 0.0188)	.39
Native Hawaiian or other Pacific Islander	−0.0402 (−0.2443 to 0.1639)	.49
Total underrepresented minorities^b^	−0.0067 (−0.0261 to 0.0128)	.28
Male	−0.0033 (−0.0140 to 0.0075)	.32
DO		
American Indian or Alaskan Native	−0.0316 (−0.0568 to −0.0063)	.03
Black or African American	−0.0053 (−0.0245 to 0.0139)	.36
Hispanic or Latino	0.0028 (−0.0128 to 0.0185)	.52
Native Hawaiian or other Pacific Islander	−0.4301 (−3.7350 to 2.8747)	.35
Total underrepresented minorities^b^	−0.0017 (−0.0203 to 0.0169)	.73
Male	−0.0029 (−0.0369 to 0.0311)	.75
DDS/DMD		
American Indian or Alaskan Native	−0.0108 (−0.1177 to 0.0961)	.71
Black or African American	−0.0014 (−0.0187 to 0.0158)	.75
Hispanic or Latino	0.0025 (−0.0193 to 0.0243)	.67
Native Hawaiian or other Pacific Islander	Not enough data	NA
Total underrepresented minorities^b^	0.0010 (−0.0081 to 0.0101)	.67
Male	−0.0124 (−0.0213 to −0.0036)	.03
PharmD		
American Indian or Alaskan Native	−0.0157 (−0.0828 to 0.0514)	.42
Black or African American	0.0002 (−0.0311 to 0.0316)	.98
Hispanic or Latino	0.0034 (−0.0070 to 0.0139)	.29
Native Hawaiian or other Pacific Islander	Not enough data	NA
Total underrepresented minorities^b^	0.0018 (−0.0175 to 0.0212)	.72
Male	0.0057 (−0.0072 to 0.0186)	.20

Abbreviations: DDS/DMD, Doctor of Dental Surgery/Medicine; DO, Doctor of Osteopathic Medicine; MD, Doctor of Medicine; NA, not applicable; PharmD, Doctor of Pharmacy; RQ, representation quotient.

^a^ The RQ is the ratio of the proportion of each subgroup to the total population of applicants, matriculants, or graduates relative to the proportion for that subgroup within the US Census population of similar age.

^b^ Underrepresented minority groups include American Indian or Alaska Native, Black or African American, Hispanic or Latino, and Native Hawaiian or Other Pacific Islander.

Regression analysis of RQ values from 2003 to 2019 collected in this study with regard to ethnicity revealed only 1 statistically significant increase (Table 2). The number of Black or African American PharmD applicants increased from 6048 to 6845 (RQ slope, 0.0187; 95% CI, 0.0007 to 0.0366; *P* = .046). Although the number of Black or African American individuals in MD programs with degrees conferred increased from 1032 to 1234, the RQ regression indicated a statistically significant decrease (RQ slope, −0.0044; 95% CI, −0.0077 to −0.0011; *P* = .03). Similarly, the number of American Indian or Alaskan Native degree earners in DO programs remained flat over the years but trended negatively (RQ slope, −0.0316; 95% CI, −0.0568 to −0.0063; *P* = .03). All others showed either no difference or decrease.

### Sex Trends

**Figure 3.**
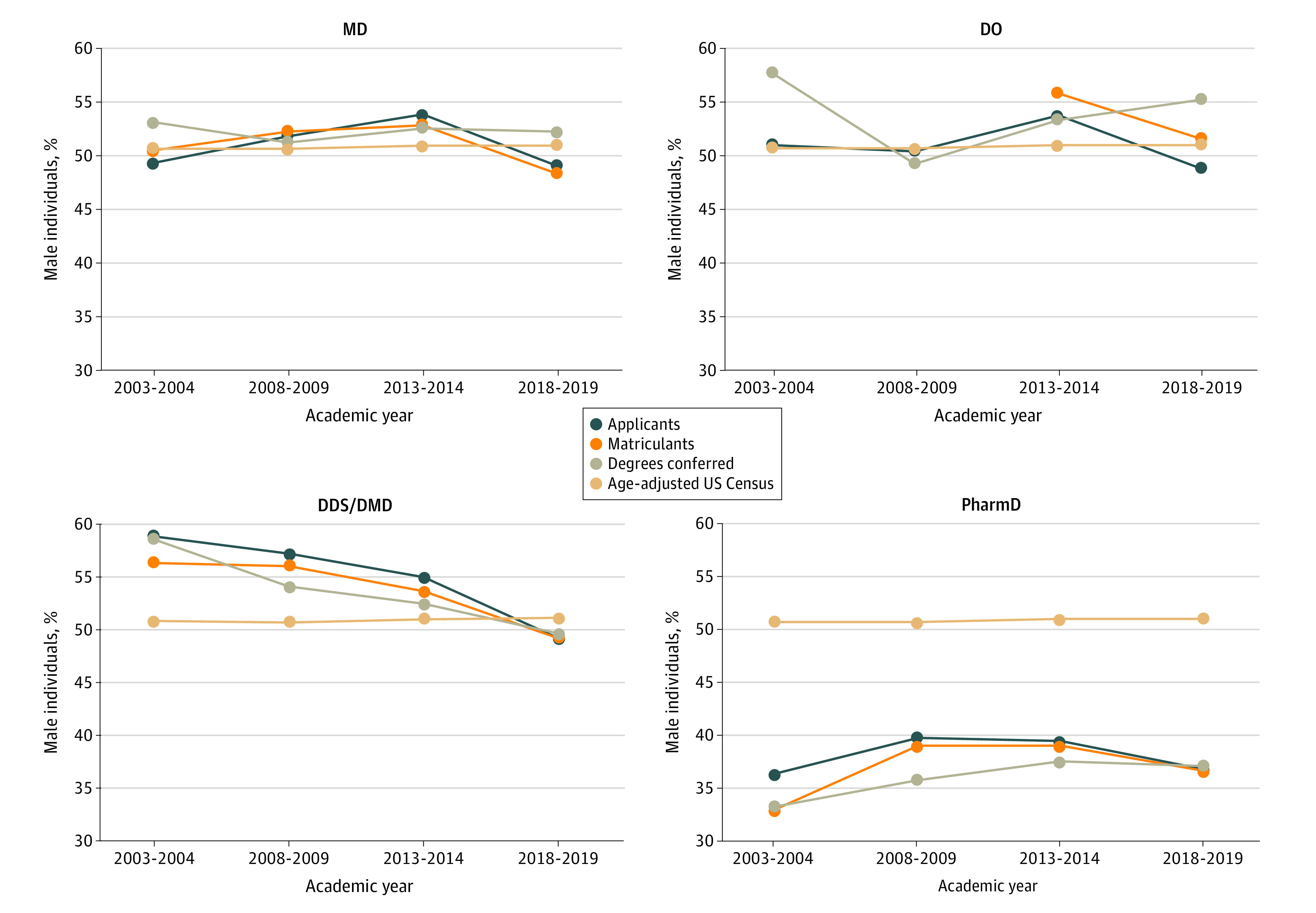
Percentages of Male Individuals in 4 Health Degree Programs, 2003-2019 Graphs show data for Doctor of Medicine (MD), Doctor of Osteopathic Medicine (DO), Doctor of Dental Surgery/Medicine (DDS/DMD), and Doctor of Pharmacy (PharmD) programs compared with the overall US population.

With regard to biological sex in the study period, all 4 health professions programs had decreased percentage of male applicants, matriculants, and degrees conferred (MD, DO, and DDS/DMD) or were below the 2020 US Census age-adjusted percentage of male population (PharmD) (Figure 3). RQ values for MD, DO, and DDS/DMD programs for applicants, matriculants, and degrees conferred over time were approximately 1.0. PharmD programs had a lower RQ (approximately 0.7) for applicants, matriculants, and degrees conferred over time (eFigure 3 in Supplement 1). Regression analysis of RQ values indicate that only DDS/DMD programs experienced a statistically significant decrease in male applicants (RQ slope, −0.0122; 95% CI, −0.0147 to −0.0098; *P* = .002), matriculations (RQ slope, −0.0109; 95% CI, −0.0196 to −0.0022; *P* = .003), and degrees conferred (RQ slope, −0.0124; 95% CI, −0.0213 to −0.0036; *P* = .03).

## Discussion

The findings of this cross-sectional study show that learners from URM groups were underrepresented across health care professions programs compared with age-adjusted US Census statistics. In DO, DDS/DMD, and PharmD programs, URM learner percentages increased, whereas in MD programs, they decreased. In terms of racial and ethnic diversity, White students are underrepresented in DDS/DMD and PharmD programs according to 2020 Census data, whereas Asian students are overrepresented across all fields. Notably, the number of Black or African American applicants to PharmD programs has increased, whereas most other URM groups have seen stable or declining numbers. Our findings align with previous literature^30,31^ showing improved female representation in MD, DDS/DMD, and DO programs. In contrast, female individuals remain overrepresented in pharmacy, possibly because of the perception that a pharmacy career is conducive to balancing professional and family life.^32^ However, sex equality might not be uniform across racial and ethnic groups.^29,31^ Research by Chapman et al^33^ and others^34,35^ underscores the influence of faculty members’ sex representation on students’ interest in a particular medical specialty.

Unlike previous studies,^23,29^ which demonstrated a decrease in URM student representation, this research spans from 2003 to 2019, covers several health professions, and depicts changes for particular racial, ethnic, and sex groups. It provides insights into students at various stages—from applicants to graduates—and reveals that underrepresentation remains a widespread concern in health professions education.

The need to diversify the health professions has been evident for decades. Persistent low representation of students and graduates from URM groups reflects structural and economic factors that also play a major role in affecting URM representation in health care programs.^36^ Recruitment strategies, holistic admissions, and pipeline programs aimed to address URM underrepresentation and have led to increasing applications, matriculations, and degree completions.^16,37,38,39,40,41^ Participants in pipeline programs report improved confidence, a sense of belonging, and views of health care as a realistic career option.^39,40^ Few pipeline programs have tracked participants longitudinally to determine their effect on application, matriculation, and degree conferral in a health professions program.^16,37,38,39,40,41^ Pipeline programs can increase the number of students from URM groups matriculating to and graduating from health professions programs.^40^ More longitudinal studies are needed to validate these findings.

Accrediting organizations for the aforementioned health education programs, such as the Liaison Committee on Medical Education (LCME), have set standards for diversity in recruitment, applicant pools, and admissions.^42,43,44,45,46,47,48,49^ Although the LCME’s 2009 diversity standards coincided with an increase in female, Black, and Hispanic matriculants, it remains unclear whether this increase was linked to the new standards.^42^ The decreasing trend currently seen in medical programs suggests that the LCME standard related to diversity is not sufficient to address the need for more diversity in the health professions programs.^50^ For medical programs, low Medical College Admission Test scores, low grade point averages, and poor science preparation have been identified as barriers to pursuing a medical career by premedical students from URM groups and to the recruitment of prospective students from URM groups.^51,52^ As noted by admissions leaders, the desire for high Medical College Admission Test scores by schools for ranking purposes and using scores for predicting future student success are barriers to efforts to increase diversity in enrollment.^53^

Despite the benefit of holistic admissions policies, lack of representation in the recruitment and admissions processes undermines potential diversity gains. Conversely, exposure during the recruitment process to students from the same background can positively influence a decision to matriculate into a program.^37^ Representation should also be present after the student is admitted and navigating the program.

Myriad efforts have sought to expand the applicant pool and promote equitable recruitment and selection processes, but few have focused on enhancing student inclusion.^50^ Research by Nwokolo et al^54^ highlights a concerning trend: a widening gap between matriculation and graduation rates for medical students from URM groups. Contributing factors include academic challenges faced by these students and a stark absence of faculty mentors from URM groups within medical schools.^54^ Hagan et al^55^ found that pharmacy had made some progress in recruiting African American or Black faculty members, although it lagged behind dental schools in terms of Hispanic faculty representation. Kamran et al^34^ highlight a misalignment between the representation of individuals from URM groups among clinical faculty and leadership roles in academic medical institutions and evolving population demographics.

Diversity efforts must extend beyond student recruitment to retaining and developing URM faculty. Lack of mentorship, an unsupportive institutional environment, and financial constraints hamper diversity improvement in health professions programs.^14,16,17,51,52,56,57,58,59,60,61^ Financial burden is a perceived barrier by students.^35,36^ Health care programs vary in number of schools, acceptance rate, length of the program, competitiveness and cost, which may affect the choice and preference for a health care major by learners from URM groups.^23^ A large number of schools, higher acceptance rates, and lower costs may attract such learners to certain professions. Finally, psychosocial factors, such as imposter syndrome, race-related stressors, and self-efficacy, may contribute to the lower number of learners from URM groups who apply to professional programs and may hinder degree completion, leading to what is termed the *leaky pipeline*.^62^

On June 29, 2023, US the Supreme Court ruled that college admissions policies incorporating race were unconstitutional, violating the Equal Protection Clause of the Fourteenth Amendment.^63^ The Court clarified that colleges and universities could still consider an applicant’s experiential qualities, including race. The long-term impact of this decision on diversity in health professions programs is unknown. In 1996, California enacted the California Civil Rights Initiative, which prohibited public institutions from considering race, ethnicity, or gender in admissions decisions. Consequently, substantial decreases in Black and Hispanic enrollment were observed at the most selective universities, whereas there were no net changes in the middle-tier institutions, and slight increases in Black and Hispanic enrollment were noted at the least selective public universities in the state.^64^

Despite new legal restrictions, various strategies can promote diversity, including scholarships, financial aid, recruitment and outreach, and pipeline and pathway programs. Data collection and race-neutral approaches, which are exempt from the US Supreme Court ruling, can also be effective. Institutional leaders should consider new policies to close the representation gap. For instance, the American Medical Association recently adopted a policy against legacy admissions.^65^

On the basis of the findings of this research, several initiatives should be considered. Programs should establish clear objectives, consistent with their accreditation standards and the recent Supreme Court ruling. Mentorship at every level of professional development and an inclusive environment are crucial.

Holistic admission criteria should be used, considering diverse skills, backgrounds, and life experiences, as well as academic credentials. Reasons for applicant selection of specific programs should inform strategies aimed at increasing the representation of students from URM groups. A comprehensive approach to support enrollment and success of students from URM groups is essential. Institutions should share effective practices and successful initiatives.

Eliminating legacy admissions can also promote equity of opportunity and diversity. The leaky pipeline phenomenon that sees students from URM groups drop out at higher rates should be addressed by providing resources and support to help students navigate their educational paths successfully. The long-term impact of initiatives should be assessed to ensure that they increase URM applications, matriculants, and graduates. Future efforts should also consider barriers that may have been previously overlooked. For example, the lack of diversity in leadership roles within these programs can affect both the choice of program and the student experience, highlighting the need for more inclusive leadership. Ultimately, an inclusive, equitable educational environment that offers continuous support to students throughout their educational journey is paramount to health care programs becoming more diverse.

Diversity barriers should be identified and actively addressed in health care education programs. The solution involves changing admission policies, enhancing support systems, and establishing holistic criteria for assessing students. Additionally, diversity should be valued as an essential component of health care delivery rather than a metric. Across different health care fields, interdisciplinary collaboration can foster knowledge sharing and best practices exchange. Such collective efforts can elevate the standard of care provided to communities, ensuring that it is high-quality and culturally competent. Despite progress, health care education still needs to be diversified. To achieve sustained success, diversity initiatives must be continually evaluated and revised.

### Limitations

This study has some limitations. First, our focus on the aforementioned health programs was influenced by the greater availability of data. Second, different methods of grouping and reporting URM populations exist between health professions, reflecting the lack of consensus in data reporting and clear definitions of ethnic groups. When schools began reporting multiple races as a standalone category, it became the third largest racial and ethnic group among medical students. This approach limits the ability to identify the identity of this group. Third, this study does not account for diversity within each URM group; for example, the Hispanic or Latino population comprises vast cultural, linguistic, and demographic variation.^56^ Fourth, this analysis masks any improvements within a health care field by region, state, and institution. Some states or regions have better representation of students from URM groups in specific health care majors.^29,66^ Future analyses should investigate whether heterogeneity exists at the regional or state levels or among other health care professions. Fifth, the collected data categorize sexual identities without accounting for the full spectrum of gender diversity. Sixth, the reporting of applicants does not exclude students who apply to multiple institutions within the same and across health majors.

## Conclusions

The relevance of this cross-sectional study cannot be overstated, given the pressing need for a health care workforce that accurately reflects the diversity of the populations it serves. Diversity initiatives have made some progress, particularly in DO, DDS/DMD, and PharmD programs. Still, underrepresentation of URM groups in these programs compared with age-adjusted US Census data serves as a sobering reminder of the significant systemic challenges. By committing to inclusivity and equity, we can move closer to a health care system that serves all individuals effectively, regardless of their background or circumstances.

## Notes

Supplement 1. **eFigure 1.** Representation Quotient Trends for Underrepresented Minorities Compared to US Census Data by Program Type **eFigure 2.** Representation Quotient by Race/Ethnicity Compared to US Census Data by Program Type in 2018-2019 **eFigure 3.** Representation Quotient Trend for Sex Compared to US Census Data by Program Type (Male)

Supplement 2.
Data Sharing Statement
