# Supplementary material for: Racial, Ethnic, and Sex Diversity Trends in Health Professions Programs From Applicants to Graduates
Source: JAMA Netw Open. 2023 Dec 28;6(12):e2347817. doi: 10.1001/jamanetworkopen.2023.47817 (PMC10755626; doi:10.1001/jamanetworkopen.2023.47817)
Supplement: Supplement 1. — eFigure 1. Representation Quotient Trends for Underrepresented Minorities Compared to US Census Data by Program Type eFigure 2. Representation Quotient by Race/Ethnicity Compared to US Census Data by Program Type in 2018-2019 eFigure 3. Representation Quotient Trend for Sex Compared to US Census Data by Program Type (Male) [file jamanetwopen-e2347817-s001.pdf]

Supplemental Online Content

Majerczyk D, Behnen EM, Weldon DJ, et al. Racial, ethnic, and sex diversity trends in health professions programs from applicants to graduates. *JAMA Netw Open*. 2023;6(12):e2347817. doi:10.1001/jamanetworkopen.2023.47817

eFigure 1. Representation Quotient Trends for Underrepresented Minorities Compared to US Census Data by Program Type
eFigure 2. Representation Quotient by Race/Ethnicity Compared to US Census Data by Program Type in 2018-2019
eFigure 3. Representation Quotient Trend for Sex Compared to US Census Data by Program Type (Male)

This supplemental material has been provided by the authors to give readers additional information about their work.

© 2023 Majerczyk D et al. *JAMA Network Open.*

eFigure 1: Representation Quotient Trends for Underrepresented Minorities Compared to US Census Data by Program Type

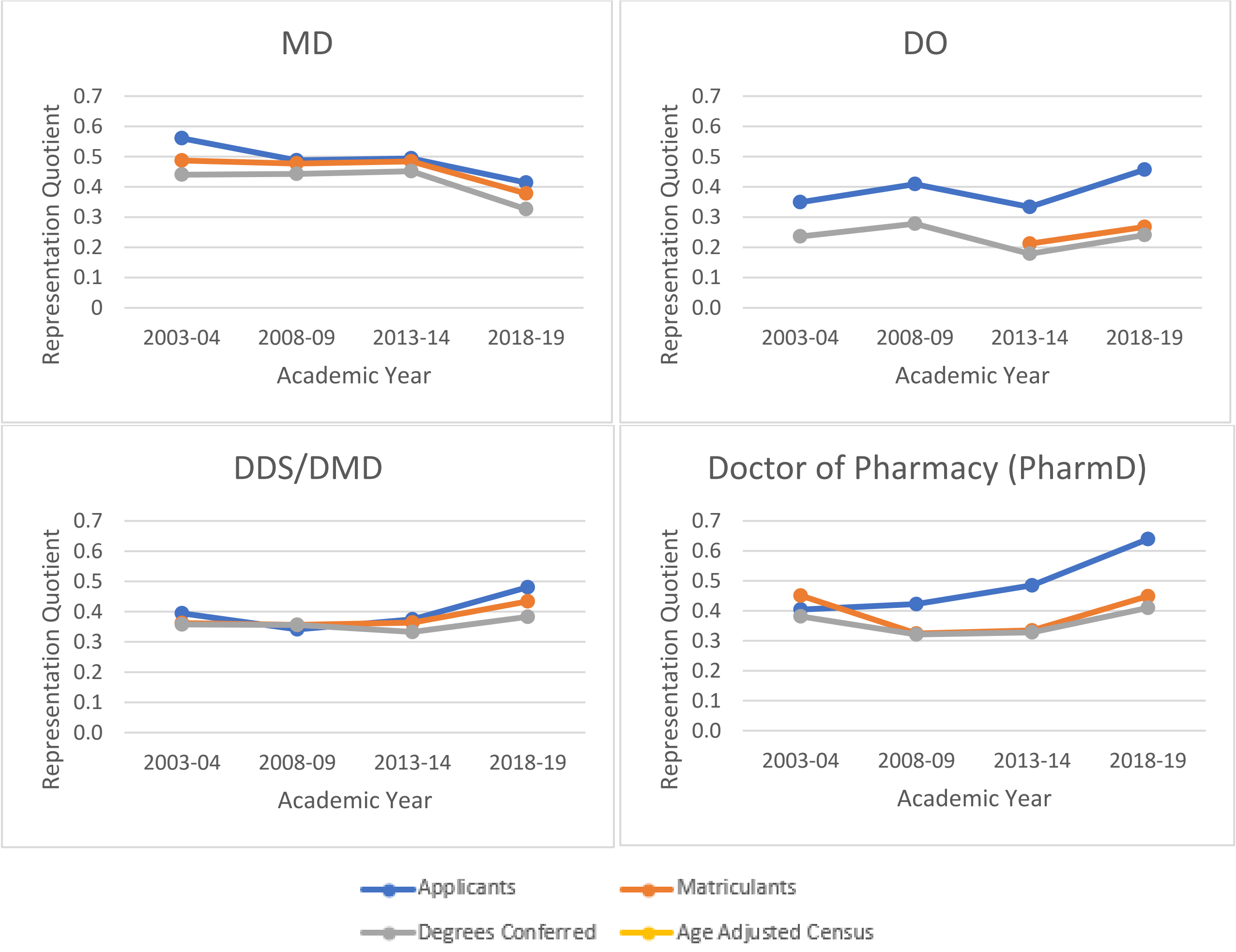

eFigure 1. Abbreviations: MD, Doctor of Medicine; DO, Doctor of Osteopathic Medicine; DDS/DMD, Doctor of Dental Surgery/Medicine; PharmD, Doctor of Pharmacy; RQ, Representation Quotient. An RQ greater than 1 indicates that a subgroup is overrepresented among respective health professions applicants (blue), matriculants (orange), and degrees conferred (grey) relative to the US population (yellow), and an RQ less than 1 indicates a subgroup is underrepresented.

© 2023 Majerczyk D et al. *JAMA Network Open.*

eFigure 2: Representation Quotient by Race/Ethnicity Compared to US Census Data by Program Type in 2018-2019

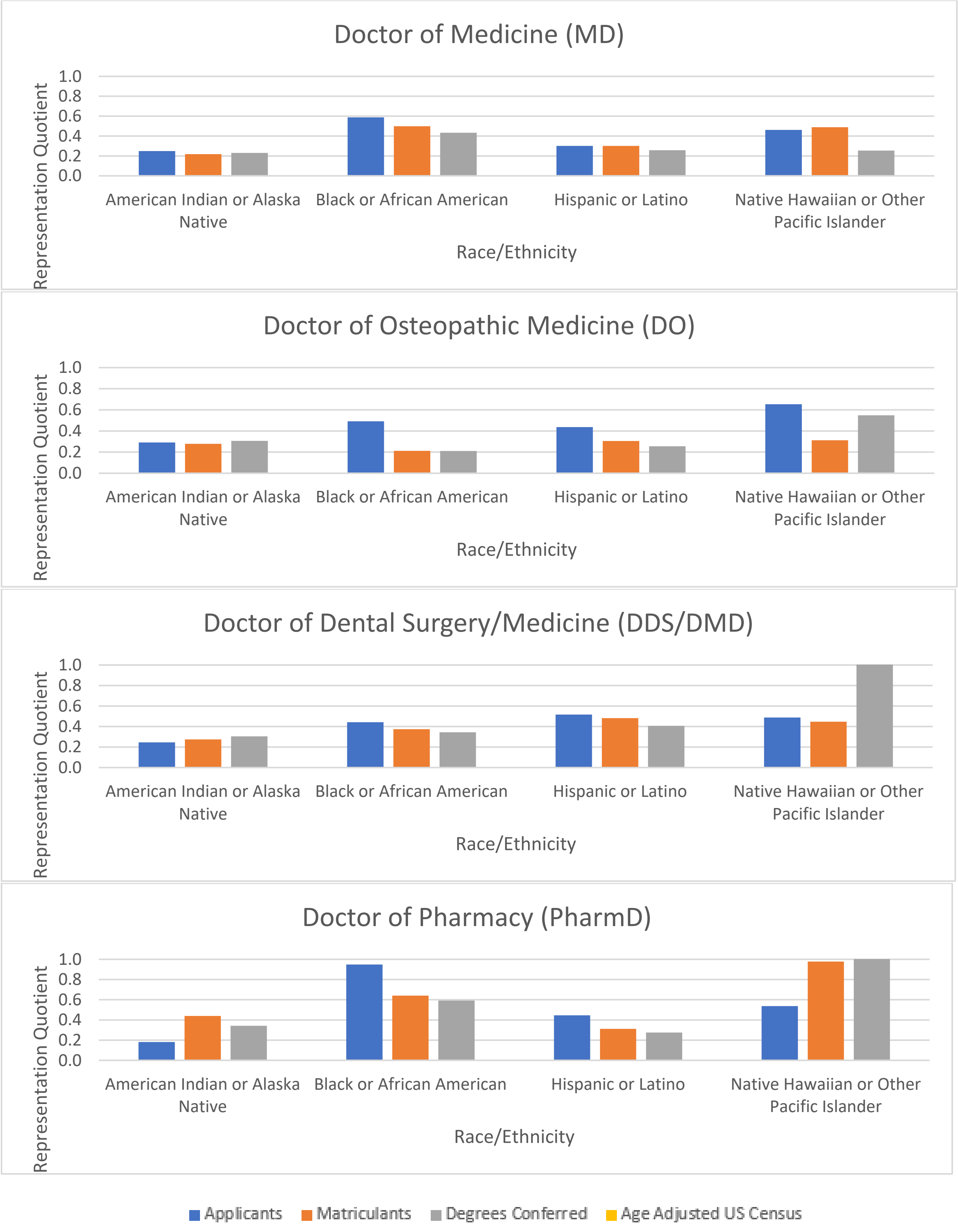

© 2023 Majerczyk D et al. *JAMA Network Open.*

eFigure 3: Representation Quotient Trend for Sex Compared to US Census Data by Program Type (Male)

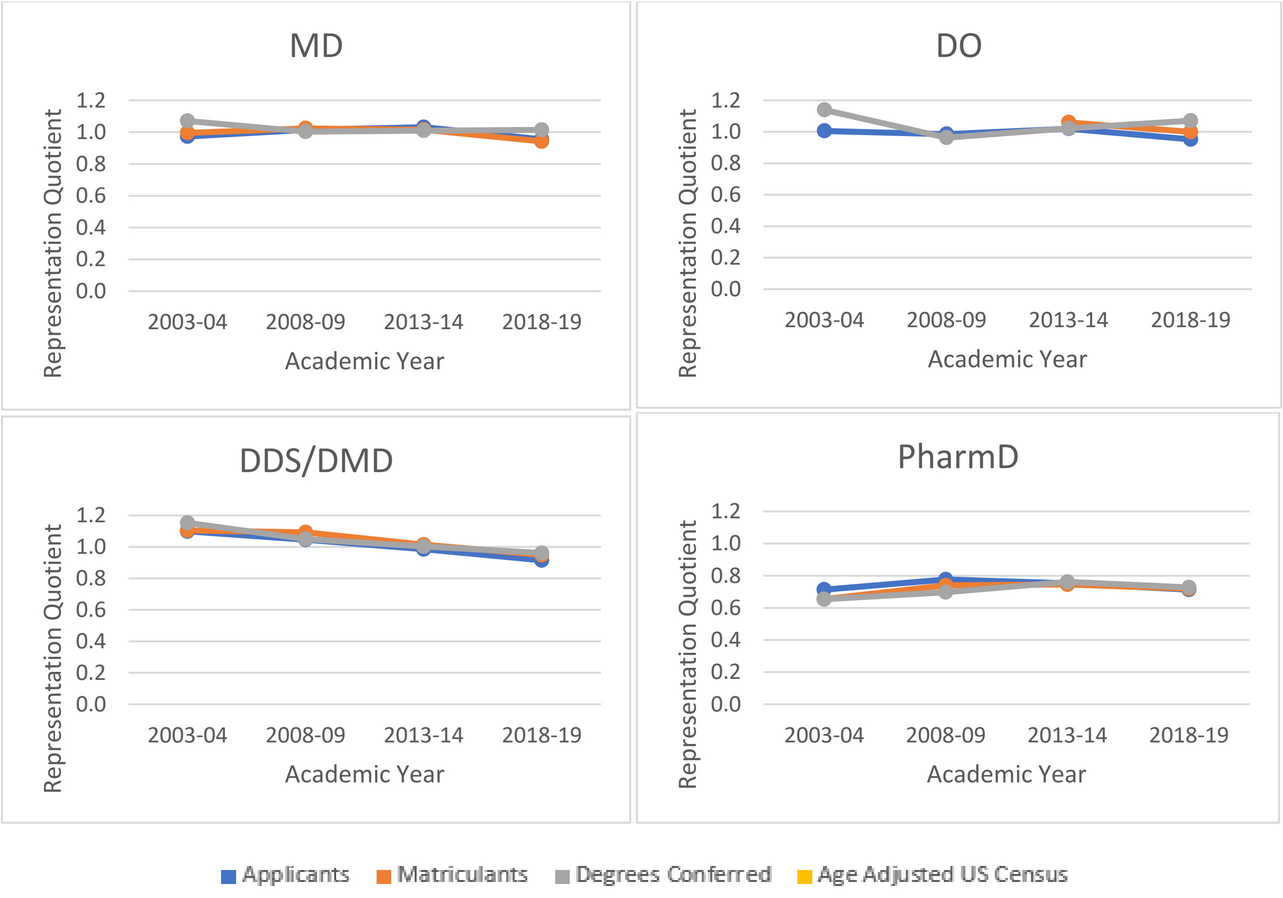

eFigure 3. Abbreviations: MD, Doctor of Medicine; DO, Doctor of Osteopathic Medicine; DDS/DMD, Doctor of Dental Surgery/Medicine; PharmD, Doctor of Pharmacy;

© 2023 Majerczyk D et al. *JAMA Network Open.*
