# Supplementary material for: Racial, Ethnic, and Sex Diversity Trends in Health Professions Programs From Applicants to Graduates
Source: JAMA Netw Open. 2023 Dec 28;6(12):e2347817. doi: 10.1001/jamanetworkopen.2023.47817 (PMC10755626; doi:10.1001/jamanetworkopen.2023.47817)
Supplement: Supplement 2. — Data Sharing Statement [file jamanetwopen-e2347817-s002.pdf]

Data Sharing Statement

Majerczyk. Racial, Ethnic, and Sex Diversity Trends in Health Professions Programs From Applicants to Graduates. *JAMA Netw Open*. Published December 15, 2023. doi:10.1001/jamanetworkopen.2023.47817

Data
Data available: Yes
Data types: Data (not involving human participants)
How to access data: daniel.majerczyk@luhs.org
When available: With publication

Supporting Documents
Document types: None

Additional Information
Who can access the data: Researchers whose proposed use of the data has been approved
Types of analyses: For any purpose
Mechanisms of data availability: With a signed data access agreement.
